# Diphenylphenoxy-Thiophene-PDI Dimers as Acceptors for OPV Applications with Open Circuit Voltage Approaching 1 Volt

**DOI:** 10.3390/nano8040211

**Published:** 2018-03-30

**Authors:** Caterina Stenta, Desiré Molina, Aurélien Viterisi, María Pilar Montero-Rama, Sara Pla, Werther Cambarau, Fernando Fernández-Lázaro, Emilio Palomares, Lluis F. Marsal, Ángela Sastre-Santos

**Affiliations:** 1 Departament d’Enginyeria Electrònica, Elèctrica i Automàtica, Universitat Rovira i Virgili, Avda. Països Catalans 26, 43007 Tarragona, Spain; caterina.stenta@urv.cat (C.S.); aurelien.viterisi@urv.cat (A.V.); mariadelpilar.montero@urv.cat (M.P.M.-R.); 2 Área de Química Orgánica, Instituto de Bioingeniería, Universidad Miguel Hernández, Avda. de la Universidad, s/n, 03203 Elche, Spain; d.molina@umh.es (D.M.); saplagar@hotmail.com (S.P.); fdofdez@umh.es (F.F.-L.); 3 Institut Català d’Investigació Química, Avda. Països Catalans 16, 43007 Tarragona, Spain; wcambarau@iciq.es (W.C.); epalomares@iciq.es (E.P.)

**Keywords:** organic solar cells, photovoltaics, perylenediimide, non-fullerene acceptor, PTB7, bulkheterojunction

## Abstract

Two new perylenediimides (PDIs) have been developed for use as electron acceptors in solution-processed bulk heterojunction solar cells. The compounds were designed to exhibit maximal solubility in organic solvents, and reduced aggregation in the solid state. In order to achieve this, diphenylphenoxy groups were used to functionalize a monomeric PDI core, and two PDI dimers were bridged with either one or two thiophene units. In photovoltaic devices prepared using PDI dimers and a monomer in conjunction with PTB7, it was found that the formation of crystalline domains in either the acceptor or donor was completely suppressed. Atomic force microscopy, X-ray diffraction, charge carrier mobility measurements and recombination kinetics studies all suggest that the lack of crystallinity in the active layer induces a significant drop in electron mobility. Significant surface recombination losses associated with a lack of segregation in the material were also identified as a significant loss mechanism. Finally, the monomeric PDI was found to have sub-optimum LUMO energy matching the cathode contact, thus limiting charge carrier extraction. Despite these setbacks, all PDIs produced high open circuit voltages, reaching almost 1 V in one particular case.

## 1. Introduction

The power conversion efficiency (PCE) of organic solar cells (OSCs) has surged in the last decade. Particularly, those made from bulk heterojunction solution-processed active layers have shown the most potential for practical applications [1]. In this respect, polymer and small molecule donors have both been the focus of significant research, leading to PCEs of over 10% when blends containing a fullerene-based acceptor [2,3,4,5] perylenediimide are used. Fullerenes such as PC_60_BM and PC_70_BM have traditionally dominated the field of solution-processed OPV. However, the high costs associated with their use have driven the quest for less synthetically-demanding acceptors. In addition to cost, the poor light absorption properties of fullerenes in the visible region of the solar spectrum limit the efficiency of the active layer to that of the donor’s fraction. Consequently, both polymers [6,7,8,9] and small molecules [10,11,12,13,14,15,16,17] have been considered as alternatives. Although polymers showed promising results initially, small-molecule non-fullerene acceptors ultimately became the most studied entities. Among them, molecules built around the coranulene, truxene, subphtalocyanine, perylenediimide cores, or linear alternating donor/acceptor-shaped molecules, have yielded the best results to date. Although the PCEs were typically lower than those of similar devices fabricated with [6,6]-phenyl-butyric acid methyl ester (PCBM), polymer/non-fullerene acceptor OSC devices have surpassed all reported records of polymer solar cells in recent tests, thus demonstrating that fullerenes are no longer an essential component in OPV [18,19,20,21,22,23,24]. Despite these encouraging results, non-fullerene acceptor materials are still yielding inconsistent results. The perylenediimide family, however, has shown more balanced performance, with several acceptors showing PCEs of over 5% [25,26,27,28,29,30,31,32], and recently, PDI-based dimers with a twisted configuration have produced a PCE of more than 8% [33]. Moreover, the important mechanisms underlying suboptimum microstructure formation in perylene-based active layers have been identified [34,35]; namely, extensive conjugation in perylene cores induces intense π–π stacking, leading to the formation of micrometer-sized acceptor domains as well as excimers in the excited state [36], both of which are detrimental to *J–V* characteristics of OSC devices. To overcome these problems, it was found that bridging two or more perylenediimide cores via their lateral ortho- or bay-positions reduced the propensity for aggregation, thus leading to the formation of an active layer with crystallites of moderate size [26,30,37,38,39,40].

In an attempt to tune the aggregation properties of PDI acceptors, herein we report on a library of PDI molecules substituted at the bay position with diphenylphenoxy groups. The perpendicular arrangement of the diphenylphenoxy group with respect to the perylene core was shown to effectively reduce the aggregation of the perylene derivatives in solution [41,42]. We extended this concept to the solid state by applying diphenylphenoxy-substituted PDIs to OSCs. We carried out the synthesis and characterization of two new PDI dimers bridged through the bay position with one and two thiophene units and applied them to bulk-heterojunction solar cells. The impact of the PDIs on the active layer microstructure was studied via X-ray and atomic force microscopy (AFM). Additionally, the optical and electronic properties of the active layers of all the PDIs were assessed via a study of non-geminate recombination kinetics, using the charge extraction/transient photovoltage (CE/TPV) method and electron and hole mobility measurements.

## 2. Results

### 2.1. Synthesis and Characterization of the PDI-Acceptors

**Scheme 1:**
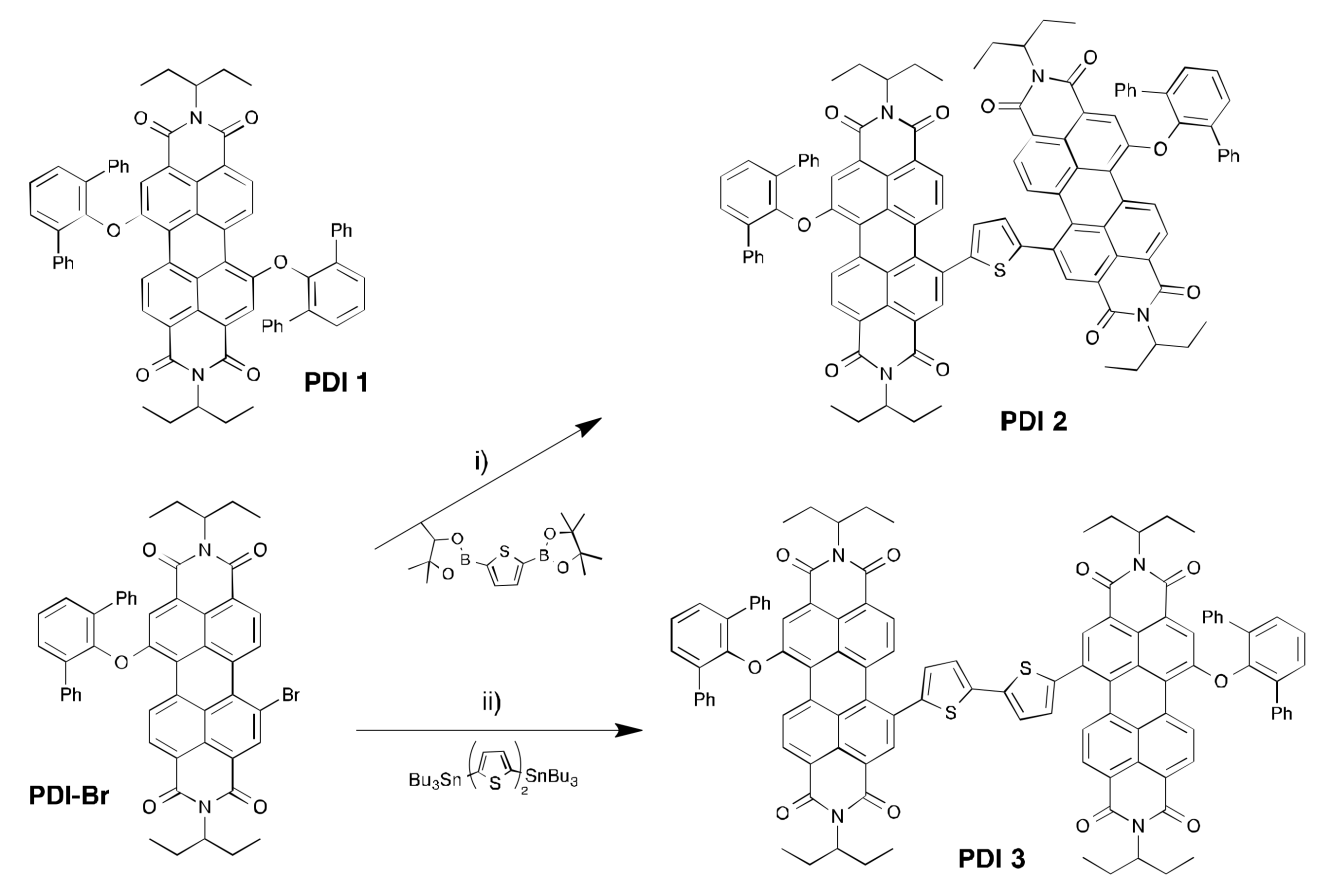
Chemical structure of PDI 1 and synthesis of PDI 2 and PDI 3. Reaction conditions: (i) Pd_2_(dba)_3_, (C_8_H_17_)_4_NBr, THF/H_2_O, 66%; (ii) Pd_2_(dba)_3_, (o-MeOPh)_3_P, Toluene, 70%.

PDI 1 was synthesized using methods reported in the literature [42]. PDI 2 was synthesized via Suzuki–Miyaura coupling from monobrominated intermediate PDI-Br (see ESI) and 2,5-*bis*(4,4,5,5-tetramethyl-1,3,2-dioxaborolan-2-yl)thiophene in a 66% yield. PDI 3 was also synthesized from PDI-Br; however, in this case, via Stille coupling with 5,5′-*bis*(tributylstannyl)-2,2′-bithiophene in a 70% yield. All derivatives were purified via flash column chromatography, and were obtained in high purity (Scheme 1).

**Figure 1:**
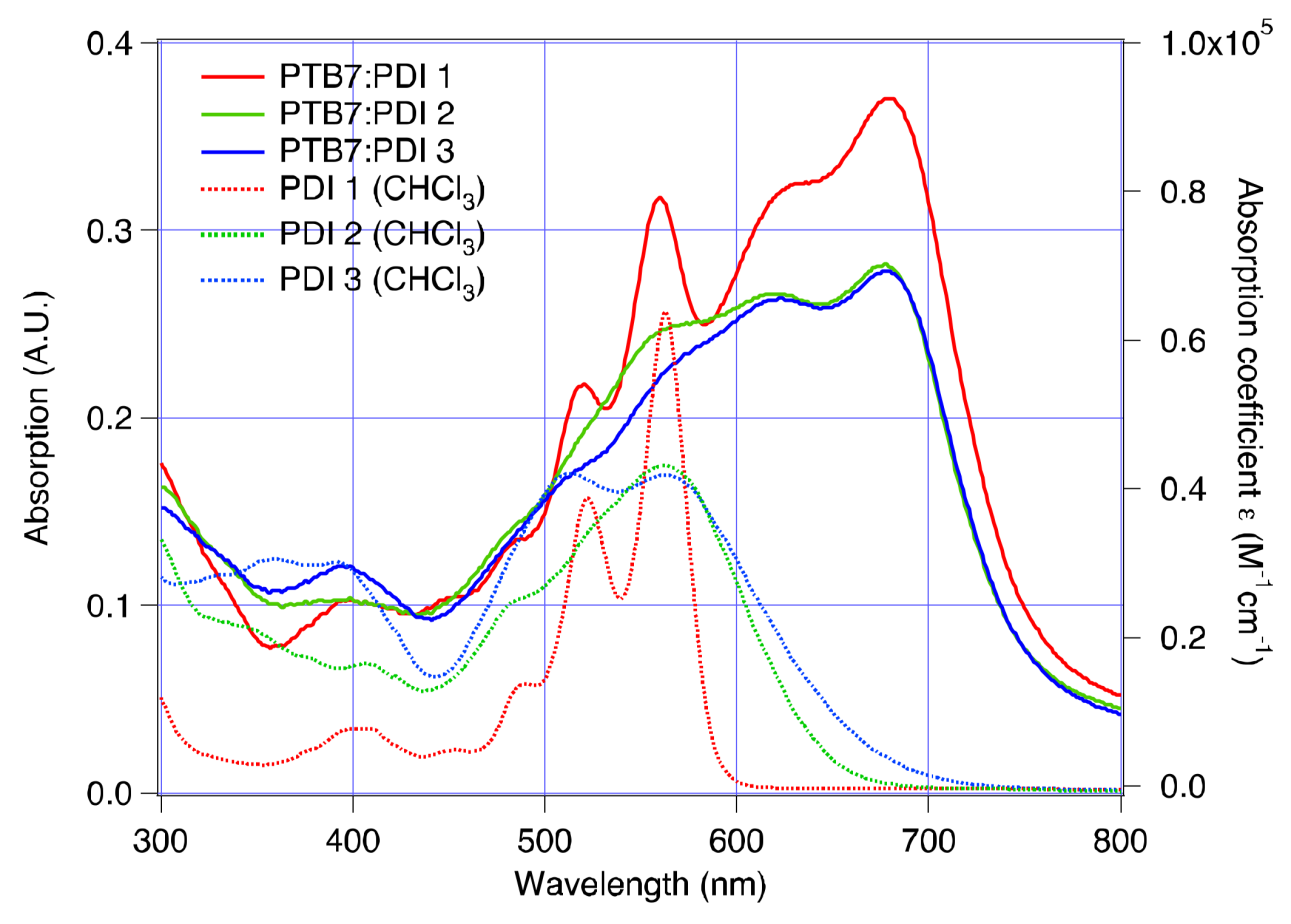
UV-Vis spectra of the PDI acceptors in chloroform (dotted line, right axis); UV-Vis spectra of active layers (PTB7:PDI) processed using deposition parameters from the fabrication of optimized devices (solid line, left axis).

**Figure 2:**
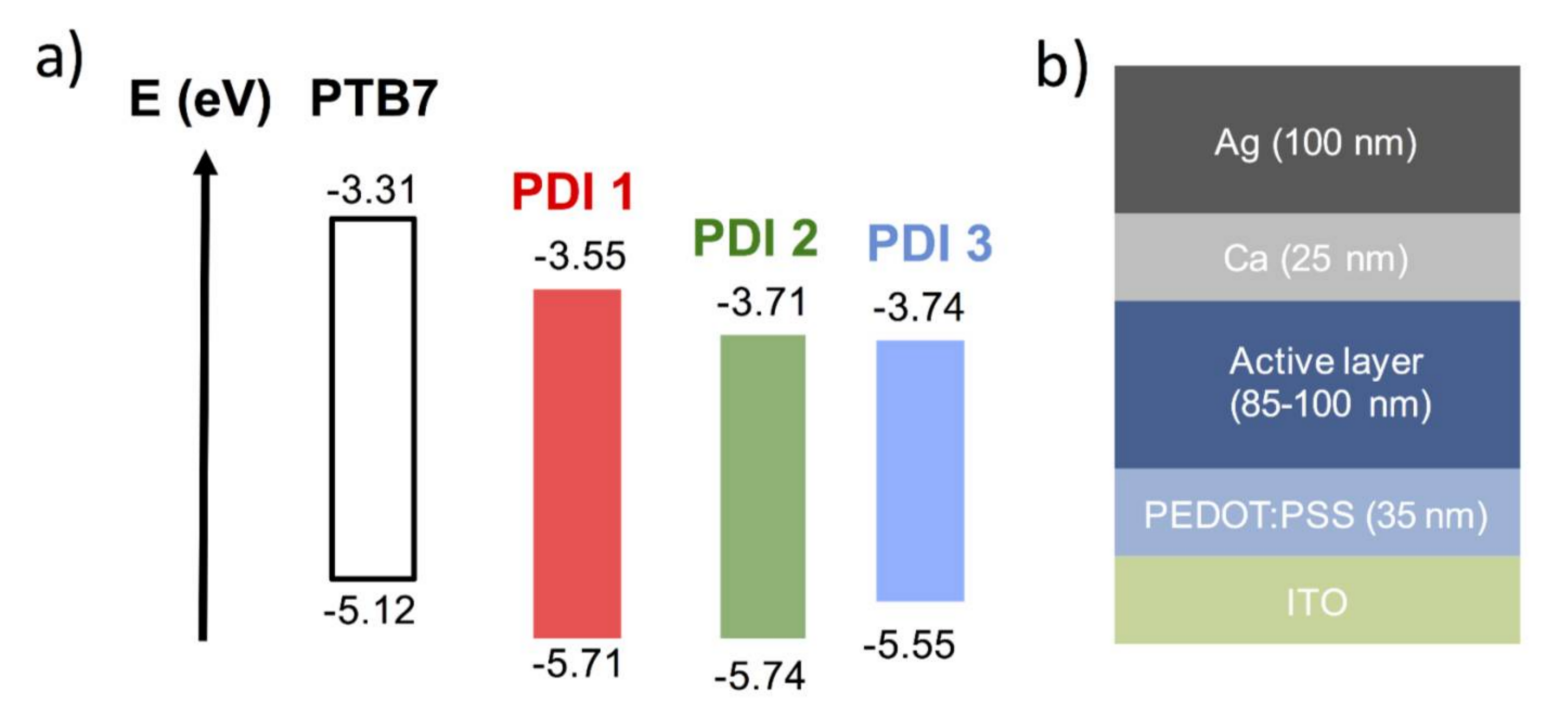
(**a**) HOMO and LUMO energy levels of the three PDIs; (**b**) Solar cell device architecture.

In solution, all three PDIs show two intense absorption bands well centered in the visible region of the absorption spectrum, with a maximum absorption coefficient in the 10^4^ range (Figure 1). Although these bands display a peak situated at an almost identical wavelength, their relative intensity varies in each PDI. The bands become broader with increasing conjugation, as seen with PDI 2 and PDI 3. When blended with PTB7 and deposited into an active layer, the PDIs are shown to extend the absorption of the active layer well in the visible region of the solar spectrum. The characteristic absorption features of both materials can be clearly seen from the absorption spectra, with absorption bands of the polymer located from 600 nm, and those of the PDIs below that value. Interestingly, with respect to the spectra, the bands belonging to the PDIs are almost unchanged when in solution. This is evidence of reduced aggregation, since a broadening of the absorption bands is usually observed in the solid state. The experimental HOMO–LUMO levels, measured via differential pulse voltammetry (see ESI) are within a similar range to those of PCBM acceptors. The LUMO levels are almost identical to those of PC_70_BM for PDI 2 and PDI 3, but significantly lower for PDI 1. The HOMO levels are slightly shifted upwards with respect to PC_70_BM (Figure 2).

### 2.2. Solar Cell Devices Fabrication and Characterization

In order to assess the photovoltaic properties of our diphenylphenoxy-substituted PDIs, we fabricated solar cell devices following standard polymer-based architectures, as shown in Figure 2.

**Figure 3:**
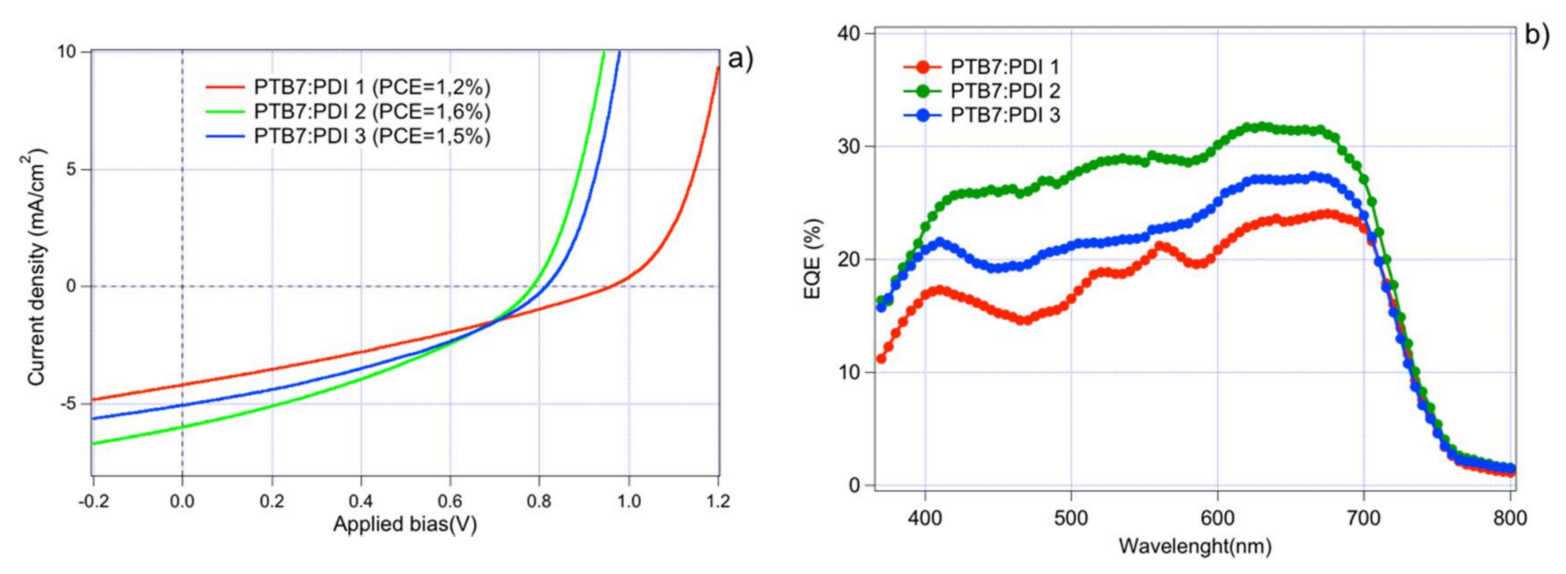
(**a**) *J*–*V* characteristics of the best performing PTB7:PDI devices recorded at 1 SUN A.M 1.5 illumination; (**b**) external quantum efficiency (EQE) spectrum of devices made from PTB7:PDI active layers.

We chose PTB7 as a candidate for the polymer donor since the benzodithiophene–thienothiophenediyl copolymer family has shown the best results in devices fabricated with PDI acceptors [26,27,28,29,30]. The three PDIs were optimized for the donor/acceptor (D/A) ratio, annealing type and additive content. PDI 2 and PDI 3 showed high solubility in chlorobenzene at the concentration used for high PCEs PTB7/PC_70_BM solar cell devices. PDI 1 had poorer solubility, and the solution of the blend was therefore diluted down to 15 mg/mL. The *J–V* characteristics of the best-performing devices are shown in Figure 3a; the main properties are shown in Table 1.

Interestingly, the best-performing devices were obtained when no annealing was applied, nor additive added, to the processing blend. Both thermal or solvent vapor annealing were found to have a detrimental effect on *J–V* characteristics, while the addition of diiodooctane (DIO) additive was found to have no positive effect. The *J–V* characteristics show modest PCEs, with limited *J*_SC_ and an unusually low fill factor (FF). The *V*_OC_, however, is seen to be higher than that of typical devices made from PCBM, approaching 1 V for PDI 1. The *J*_SC_ is higher with dimers than with the mono-adduct, while the *V*_OC_ is lower. Amongst the two dimers, the monothiophene-bridged PDI 2 was shown to yield the highest efficiency, although that of PDI 3 was very similar.

The *J*_SC_ trend is corroborated by the external quantum efficiency (EQE) spectrum of PDI-made devices, as seen in Figure 3b. Importantly, the PDI acceptors are seen to contribute significantly to photon conversion efficiency at lower wavelengths (500 to 600 nm). The EQE over the 350–500 range is, however, slightly lower than that of the remaining wavelengths as a result of the limited absorption of the active layers in that region of the spectrum (Figure 1).

### 2.3. Morphologival Characterization

To assess the impact of the PDIs whereby the formation of over-sized crystalline domains is avoided, we conducted a morphological study using X-ray diffraction and AFM microscopy techniques. Thin films of active layers were deposited on Pedot:PSS-covered glass substrates and monocrystalline Silicon (001) substrates in identical conditions to those of solar cell devices. Bragg–Brentano (theta–theta) diffractograms and grazing incidence X-ray diffraction (GIXRD) 2D area images of active layers made from each PDI were recorded in identical conditions as those previously described for PTB7/PCBM blends (see ESI) [43,44]. Unfortunately, no diffraction peaks could be detected using either technique, indicating that the PDI does not form crystalline domains in the active layer, and that it does not promote the formation of crystalline domains of polymer.

**Figure 4:**
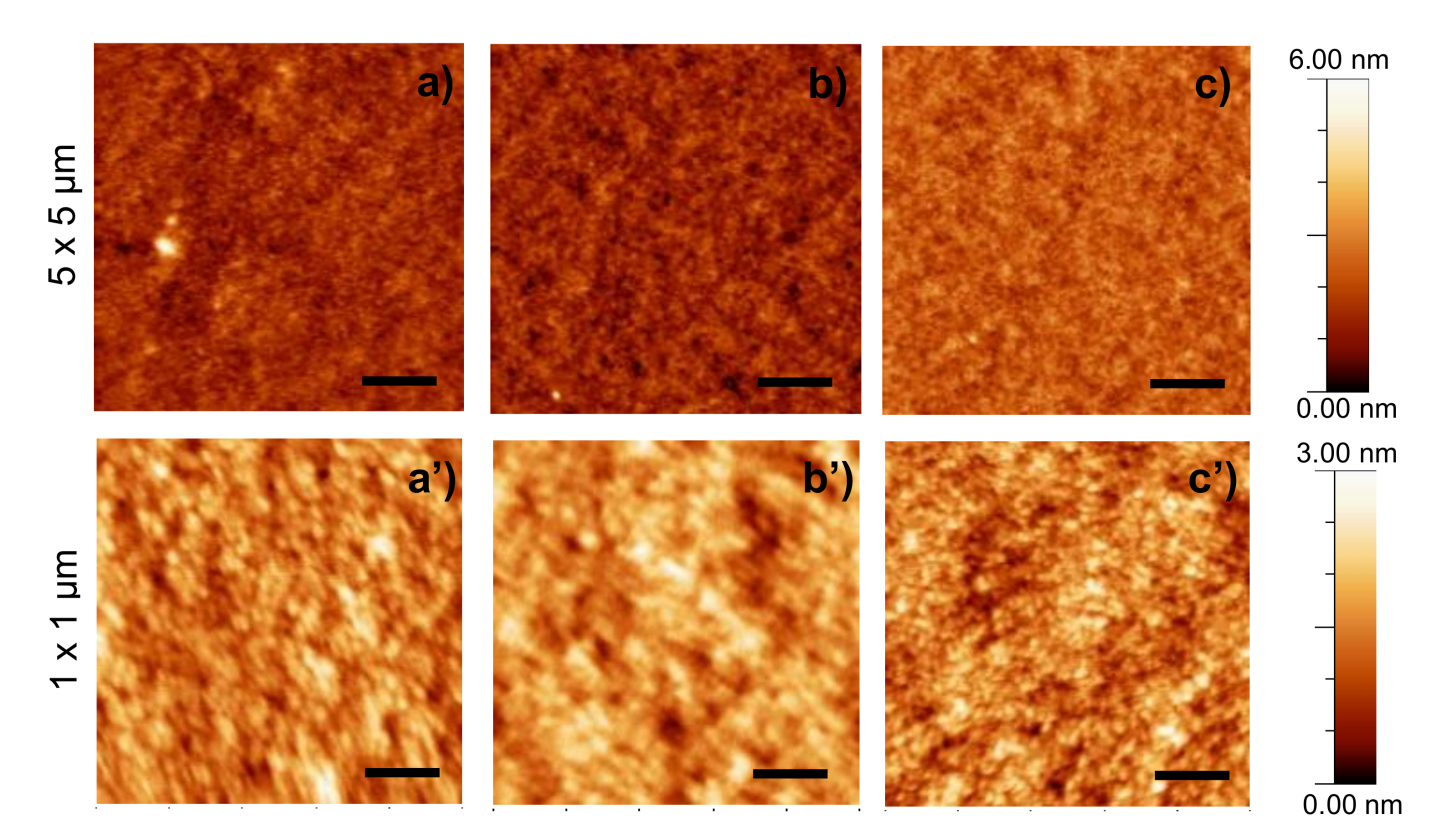
AFM images of the active layers made from PTB7 and PDI blends in conditions of optimized solar cell devices. (**a**,**a’**) PTB7:PDI 1 (1:1); (**b**,**b’**) PTB7:PDI 2 (1:1); (**c**,**c’**) PTB7:PDI 3 (1:1). Scale bar corresponds to 1 μm in the top pictures, and 200 nm in the bottom pictures.

The AFM images recorded on active layers from each PDI are shown in Figure 4. As shown, all PDIs demonstrate a marked tendency to form very smooth surfaces. This corroborates the results from XRD, in which all PDIs were shown to form amorphous layers when deposited onto thin films. This is further exemplified by the RMS roughness values of the active layers, as well as the peak-to-peak height, both of which are below the average of standard PTB7-based OSCs (Table S3, ESI). The rather featureless phase images corroborate the lack of phase segregation which could be deduced from the topography images (Figure S8, ESI).

### 2.4. Electrical and Photophysical Characterization

To link the morphology data with the photophysical properties of the devices, we carried out electron mobility measurements on hole-only and electron-only devices fabricated under identical conditions as those of OSC devices. We used the Mott–Gurney equation on the space charge-limited current (SCLC) region of the hole- and electron-only devices. The *J–V* characteristics were measured in the dark to calculate the zero-field electron mobility of the devices (see ESI) [45,46]. Our analysis shows that hole mobility is very similar in devices made from any of the PDIs ranging from 3.4 to 4.3 × 10^−4^ cm^2^/V·s. However, electron mobility is up to three orders of magnitude lower that of hole mobility in all cases, creating a strong imbalance in carrier mobility. The effect on devices’ characteristics is expounded in the section below.

**Figure 5:**
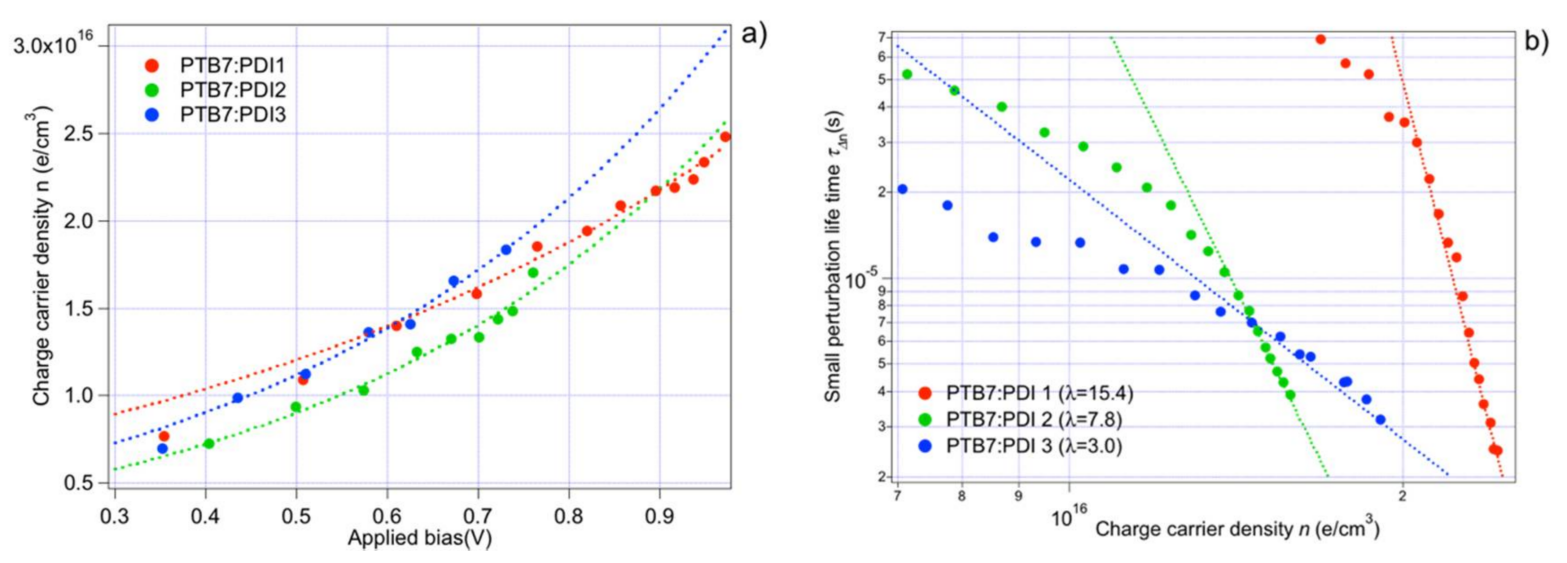
(**a**) Charge density (n) as a function of the open circuit voltage determined from CE measurement. The curves are fitted to an exponential growth of the form *n* = *n*_0_*e*^(*γVoc*)^ (dotted line) whose parameters are reported in Table 2; (**b**) Carriers’ lifetime measured using TPV as a function of device open circuit voltage. The curves are fitted to a power decay of the form *τ*Δ*n* = *τ*Δ*n*_0_·*n*^−^*^λ^* (dotted line), whose parameters are reported in Table 2.

The large difference in *V*_OC_ between PDI 1 and the other two PDIs cannot be attributed solely to frontier energy orbital energetics (the difference between the LUMO energy level of the acceptor and the HOMO level of the donor). However, as previously shown with similar devices, the experimental difference in *V*_OC_ is more likely to be attributable to non-geminate recombination kinetics [47,48,49]. In order to identify how the latter contributes to *V*_OC_, and to some extent to the shape of the *J–V* characteristics, a qualitative recombination study was carried out using a well-established charge extraction and transient photovoltage method (see ESI for experimental details). We used CE measurements to estimate the average charge density under open circuit conditions. Figure 5a shows the plot of the charge density (n) vs. open circuit voltage obtained from CE for all the devices, where n was corrected for electrode capacitance. The data is consistent with a charge density of similar magnitude in all measurements, reaching about 2 × 10^16^ charges per cm^3^, similar to what was reported earlier for such devices. [50,51,52,53] The total charge is seen to increase exponentially with the applied bias, a feature linked to an accumulation of charges in the bulk of the device. The plots were fitted to single exponentials according to Equation (1), and were analogous to the splitting of the quasi-Fermi levels in intrinsic semiconductors, where the value of γ (see Table 2) is indicative of an exponential tail of trap states extending into the band gap of the active layer [54,55,56,57].

(1) $$n=n_{0}e^{\gamma Voc}$$

(2) $$\frac{dn}{dt}=-kn^{\phi }$$

(3) $$\tau_{\Delta n}=\tau_{\Delta n_{0}}n^{-\lambda }$$

The plots in Figure 5a and Figure S14 were combined, as shown in Figure 5b, and used to determine the overall order of recombination, defined by Equation (2); this can be approximated to *φ* = *λ* + 1 under our TPV experimental conditions (Δ*n* << *n*) [58]. The parameter *λ* is obtained experimentally by fitting the curve of the small perturbation carrier life time *τ*Δ*n* vs. n to a power law according to Equation (3).

Interestingly, the recombination order is seen to vary very significantly from device to device. For PDI 1 in particular, it shows an extremely high value (*φ* = 16.5). Such values, as opposed to a value of 2 (which would be expected in a strictly bimolecular recombination process), have been measured several times in earlier studies [56,57,58,59,60,61,62,63,64]. Importantly, recombination life times measured through CE/TPV correspond to total charge carrier recombination; therefore, it is difficult to attribute the experimental reaction order (*φ*), obtained using this method, to a specific recombination mechanism. Indeed, it has been shown that, especially in thin active layers as in our case, surface recombination or doping can have a very significant influence on the apparent recombination order [65].

**Figure 6:**
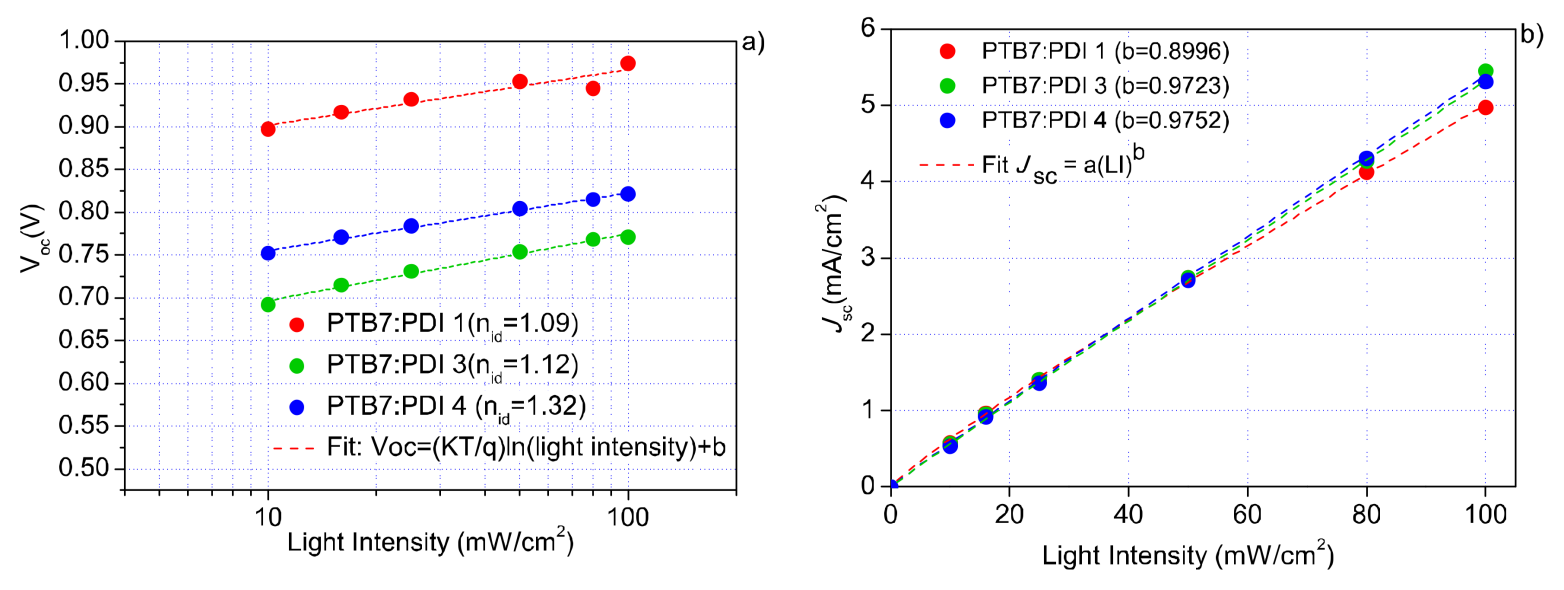
(**a**) Open circuit voltage vs. light intensity plot for all PDIs; (**b**) Short circuit current density vs. light intensity plot for all PDIs.

To gain more insight into this matter, we calculated the ideality factor (n_id_) of all devices by fitting the *V*_OC_ vs. light bias plot to a logarithmic function as depicted in Figure 6a. The data for n_id_ is in the range of expected values for thin devices (thickness < 100 nm) where surface recombination is significant. This is consistent with the rather high recombination orders observed experimentally. The case of the extremely high recombination order in PDI 1 is consistent with the presence of a stronger inhomogeneity of carrier concentration across the thickness of the active layer [65]. This is confirmed both by the lower n_id_ than the other two PDIs, and the *J*_SC_ vs. Light intensity plots (Figure 6b), which shows a sub-unity power law dependency for PDI 1. 

**Figure 7:**
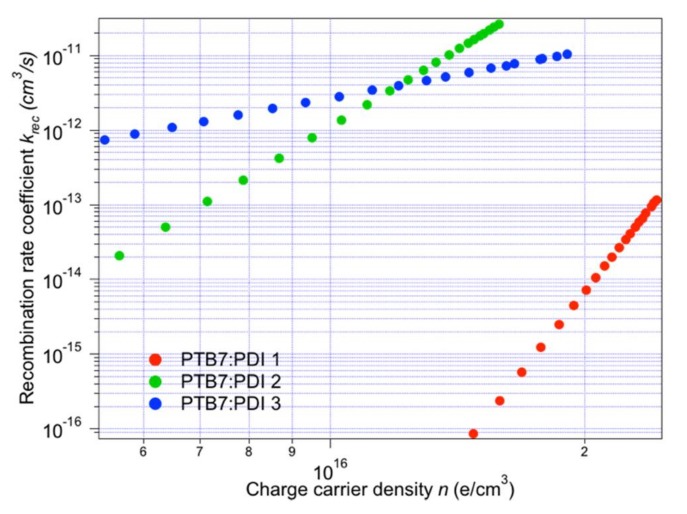
Recombination coefficient, *K_rec_*, as a function of charge density.

This non-linear behavior is indicative of the formation of a space charge region which is consistent with carrier dynamics dominated by surface recombination. This space charge region likely originates from the carrier mobility imbalance to a large extent, as well as from the sub-optimum HOMO energy level of PDI 1, −3.5 eV vs. −3.7 eV for PCBM, which creates a barrier to electron extraction at the cathode. Finally, despite these additional losses, the recombination coefficient *K_rec_*, corresponding to a charge-dependent non-geminate recombination coefficient as defined by Equations (4) and (5) [47,54,58], follows the trend observed with the *V*_OC_, indicating that non-geminate recombination plays a major role in determining the *V*_OC_ of the devices (Figure 7).

(4) $$\frac{dn}{dt}=-k_{rec}\left(n\right)n^{2}$$

(5) $$k_{rec}\left(n\right)=\frac{n^{\lambda -1}}{\left(\lambda +1\right)\tau_{\Delta n_{0}}n_{0}^{\lambda }}$$

The small perturbations in recombination kinetics exhibited by devices made from our PDIs are seen to be up to one order of magnitude slower than those reported for similar devices made from PCBM as an acceptor [43,50,53]. As a result, in devices made from PDIs, the *V*_OC_ reached a value up to 250 mV higher than that of devices made from PC_70_BM, despite the fact that the latter has an almost identical LUMO energy level to that of PDI 2 and PDI 3.

Despite the fact that PDIs lead to higher *V*_OC_ than state-of-the-art devices, the *J*_SC_ and shunt resistance are lower than average (Table 2). It is likely that the amorphous nature of the active layers induces the formation of highly mixed D/A domains with poor phase segregation. This, in turn, presumably results in a high rate of geminate recombination limiting the *J*_SC_, and a slow rate of charge transport (as seen by the low electron mobility), resulting in a low shunt resistance.

## 3. Conclusions

In the present study, we have demonstrated that the functionalization of PDI cores at the bay position with diphenylphenoxy groups leads to completely amorphous active layers when blended with PTB7. The perpendicular arrangement of the diphenylphenoxy groups with respect to the perylenediimide’s plane likely impedes intermolecular π–π stacking between the conjugated cores to such an extent that the formation of crystalline domains in the solid state is suppressed. This results in devices with lower-than-average electron mobility when PDIs are used as acceptors instead of PC_70_BM, as well as to significantly slower recombination kinetics. Consequently, these devices exhibit a significantly higher *V*_OC_ than PCBM-based devices. However, additional losses occur due to the lack of phase segregation between donor and acceptor phases. Surface recombination becomes significant and the electron and hole mobility imbalance induce a decrease in charge extraction efficiency. This leads to *J*–*V* characteristics with moderate *J*_SC_, and a typically low fill factor. Work is underway involving the substitution of the diphenylphenoxy at bay position for less sterically demanding groups.

## Figures and Tables

**Table 1:** Organic solar cell (OSC) device parameters.

Acceptor	AL Thickness (nm)	*J*_SC_ (mA/cm^2^)	*V*_OC_ (mV)	FF	PCE (%)
PDI 1	100	4.20	0.960	0.29	1.20
PDI 2	90	5.98	0.784	0.35	1.61
PDI 3	85	5.04	0.814	0.36	1.54

**Table 2:** Values of recombination parameters and hole and electron mobility derived from charge extraction/transient photovoltage (CE/TPV) measurements and SCLC.

Acceptor	*n*_0_	*γ*	*β*	*φ*	μh (cm^2^/V)	μe (cm^2^/Vs)	R_S_ (Ω/cm^2^)	R_shunt_ (Ω/cm^2^)
PDI 1	5.7 × 10^15^	1.47	25.7	16.4	3.4 × 10^−4^	2.75 × 10^−7^	28.3	311.6
PDI 2	3.0 × 10^15^	2.21	13.7	8.8	4.3 × 10^−4^	6.02 × 10^−7^	7.5	255.7
PDI 3	3.8 × 10^14^	2.14	5.2	4.0	4.1 × 10^−4^	9.53 × 10^−7^	7.05	327.9

## Supplementary Materials

Detailed experimental procedures and additional characterization data are available online at http://www.mdpi.com/2079-4991/8/4/211/s1.
