# Supplementary material for: Diphenylphenoxy-Thiophene-PDI Dimers as Acceptors for OPV Applications with Open Circuit Voltage Approaching 1 Volt
Source: Nanomaterials (Basel). 2018 Mar 30;8(4):211. doi: 10.3390/nano8040211 (PMC5923541; doi:10.3390/nano8040211)
Supplement: Supplementary file 1 [file nanomaterials-08-00211-s001.pdf]

Supplementary Materials

Diphenylphenoxy-Thiophene-PDIs acceptors for OPV applications with open circuit voltage approaching 1 volt.

Caterina Stenta, Desiré Molina, Aurelien Viterisi, María Pilar Montero-Rama, Sara Pla, Werther Cambarau, Fernando Fernández-Lázaro, Emilio Palomares, Lluis F. Marsal, Ángela Sastre-Santos **

General Remarks on Experimental Data

Synthetic Procedures: All chemicals were reagent-grade, purchased from commercial sources, and used as received, unless otherwise specified. Column chromatography: SiO_2 (40-63 µm) TLC plates coated with SiO_2 60F254 were visualized by UV light. NMR spectra were measured with a Bruker AC 300. UV-vis spectra were recorded with a Helios Gamma spectometer at 298 K in $CHCl_3$, and IR spectra and a Nicolet Impact 400D spectrophotomete in a KI matrix. Fluorescence spectra were recorded with a Perkin Elmer LS 55. Mass spectra were obtained from a Bruker Microflex matrix-assisted laserdesorption/ionization time of flight (MALDI-TOF).

Synthesis Procedures and Characterisation Data

PDI 2: In a 100 ml round-bottom flask were introduced PDI 4 (513 mg, 0.60 mmol), bis(4,4,5,5-tetramethyl-1,3,2-dioxaborolan-2-yl)thiophene (124 mg, 0.37 mmol), $Pd_2(dba)_3$ (36 g, 0.04 mmol) and tetraoctylammonium bromide (21 mg, 0.04 mmol). Then the solvents were added subsequently, THF (40 mL) and K_2CO_3 2M (2 ml). The reaction mixture was stirred for 3 h at 80 ºC under an atmosphere of argon. After cooling to room temperature, the crude product was extracted with dichloromethane and washed with water. The solvent was removed under reduced pressure and the crude product was purified by silica gel column chromatography (SiO_2, dichloromethane:acetone 25:1) to yield **PDI 2** (398 mg, 66 %) as a purple solid. ^{1}H NMR (300 MHz, $CDCl_3$, 300 K, δ): δ =

9.11 (2H, d, *J*=8.4 Hz, 2xPDI-*H*), 8.74 (2H, s, 2xPDI-*H*), 8.59 (2H, d, *J*=8.4 Hz, 2xPDI-*H*), 8.42 (2H, d, J=8.1 Hz, 2xPDI-*H*), 8.13 (2H, d, J=8.1 Hz, 1xPDI-*H*), 7.86 (2H, s, 2xPDI-*H*), 7.50 (6H, s, 6xAr-*H*), 7.39-7.35 (10H, m, 8xAr-H+2xthiophene-H), 6.99-6.88 (12H, m, 12xAr-*H*), 5.17-5.06 (2H, m, 2xPDI-C*H*(CH_2CH_3)$_2$), 4.87-4.77 (2H, m, 2xPDI-C*H*(CH_2CH_3)$_2$), 2.39-2.24 (4H, m, 2xPDI-CH(C*H*HCH_3)$_2$), 2.11-1.92 (8H, m, 4xPDI-CH(CH*H*CH_3)$_2$), 1.84-1.73 (4H, m, 2xPDI-CH(C*H*HCH_3)$_2$), 0.97 (12H, t, *J*=7.5 Hz, 2xPDI-CH($CH_2C\mathit{H_3}$)$_2$), 0.73 ppm (12H, t, *J*=7.5 Hz, 2xPDI-CH($CH_2C\mathit{H_3}$)$_2$); ^{13}C NMR (75 MHz, $CDCl_2$-$CDCl_2$): δ = 164.0, 163.9, 163.6, 163.2, 155.7, 148.0, 146.5, 137.2, 135.8, 135.1, 133.7, 133.3, 132.6, 131.2, 131.0, 129.8, 128.8, 128.7, 128.4, 128.3, 128.0, 127.8, 127.3, 126.4, 123.9, 123.3, 122.4, 122.2, 121.8, 121.0, 120.7, 57.7, 57.5, 25.0, 24.8, 11.4, 11.1 ppm; IR (KBr) *ν*: 3442, 3056, 2961, 2874, 1698, 1658, 1589, 1505, 1458, 1405, 1327, 1261, 1191, 1073, 903, 859, 811, 754, 699, 582, 408 cm^{-1}; UV/Vis (chloroform), λ_{max} (log *ε*): 408 (4.22), 562 nm (4.64); MS (MALDI-TOF): m/z: calcd for $C_{108}H_{84}N_4O_{10}S$: 1626.591 [M]$^-$; found: m/z: 1628.543 [M]$^-$.

PDI 3: In a dry 25 ml round-bottom flask, PDI 4 (100 mg, 0.12 mmol), 5,5′-bis(tributylstannyl)-2,2′-bithiophene (36 mg, 0.05 mmol), tris(dibenzylideneacetone)dipalladium(0) ($Pd_2(dba)_3$) (4.3 mg, 4.7x10^{-3} mmol) were introduced, and tris(2-methoxyphenyl)phosphine ((*o*-MeOPh)$_3$P) (6.6 mg, 0.02 mmol) was stirred in dry toluene (1 mL) under argon for 48 h at 110 ºC. The solvent was removed under reduced pressure and the crude product was purified by silica gel column chromatography (SiO_2, chloroform) to yield **PDI 3** (60 mg, 70 %) as a shiny purple solid. ^{1}H NMR (300 MHz, $CDCl_3$, 300 K, *δ*): *δ* = 9.16 (2H, d, *J*=8.4 Hz, 2xPDI-*H*), 8.70 (2H, s, 2xPDI-*H*), 8.58 (2H, d, *J*=8.4 Hz, 2xPDI-*H*), 8.35 (2H, d, *J*=8.2 Hz, 2xPDI-*H*), 8.14 (2H, d, *J*=8.2 Hz, 1xPDI-*H*), 7.92 (2H, s, 2xPDI-*H*), 7.55-7.52 (6H, m, 6xAr-*H*), 7.45-7.42 (8H, m, 8xAr-*H*), 7.21 (2H, d, *J*=3.7 Hz, 2xthiophene-*H*), 7.16 (2H, d, *J*=3.7 Hz, 2xthiophene-*H*), 7.09-7.01 (12H, m,

12xAr-*H*), 5.14-5.04 (2H, m, 2xPDI-C*H*(CH_2CH_3)$_2$), 4.98-4.87 (2H, m, 2xPDI-C*H*(CH_2CH_3)$_2$), 2.37-2.22 (4H, m, 2xPDI-CH(C*H*HCH_3)$_2$), 2.20-2.07 (4H, m, 2xPDI-CH(CH*H*CH_3)$_2$), 2.03-1.79 (8H, m, 2xPDI-CH(C*H*HCH_3)$_2$), 0.95 (12H, t, *J*=7.4 Hz, 2xPDI-CH(CH_2C*H*$_3$)$_2$), 0.86 ppm (12H, t, *J*=7.4 Hz, 2xPDI-CH(CH_2C*H*$_3$)$_2$).; ^{13}C NMR (75 MHz, $CDCl_2$-$CDCl_2$): δ = 164.3, 164.2, 164.0, 163.5, 155.9, 148.0, 143.9, 139.0, 137.5, 136.0, 134.0, 133.7, 133.1, 131.7, 131.3, 129.9, 129.1, 128.6, 128.4, 128.1, 127.5, 126.7, 125.9, 124.2, 123.5, 122.4, 122.2, 122.0, 121.0, 120.9, 57.9, 57.7, 25.2, 25.1, 11.6, 11.4 ppm; IR (KBr) ν: 3432, 2961, 2928, 2874, 1698, 1659, 1596, 1505, 1459, 1405, 1327, 1261, 1193, 1074, 903, 860, 812, 754, 700, 408 cm^{-1}; UV/Vis (chloroform), λ_{max} (log ε): 358 (4.48), 392 (4.48), 514 (4.62), 562 nm (4.62); MS (MALDI-TOF): m/z: calcd for $C_{112}H_{86}N_4O_{10}S_2$: 1709.571 [M-H]$^-$; found: m/z: 1709.524 [M-H]$^-$.

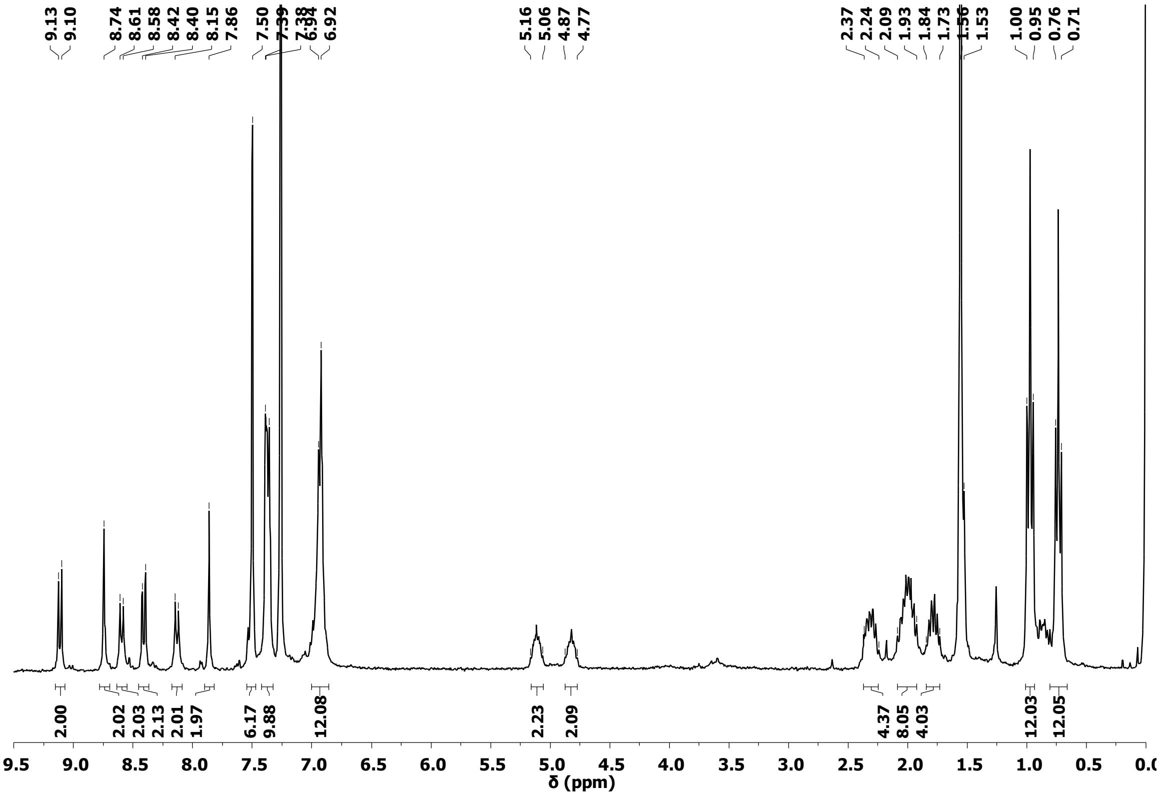

Figure S1. ^{1}H-RMN ($CHCl_3$, 25°C) spectrum for **PDI 2**.

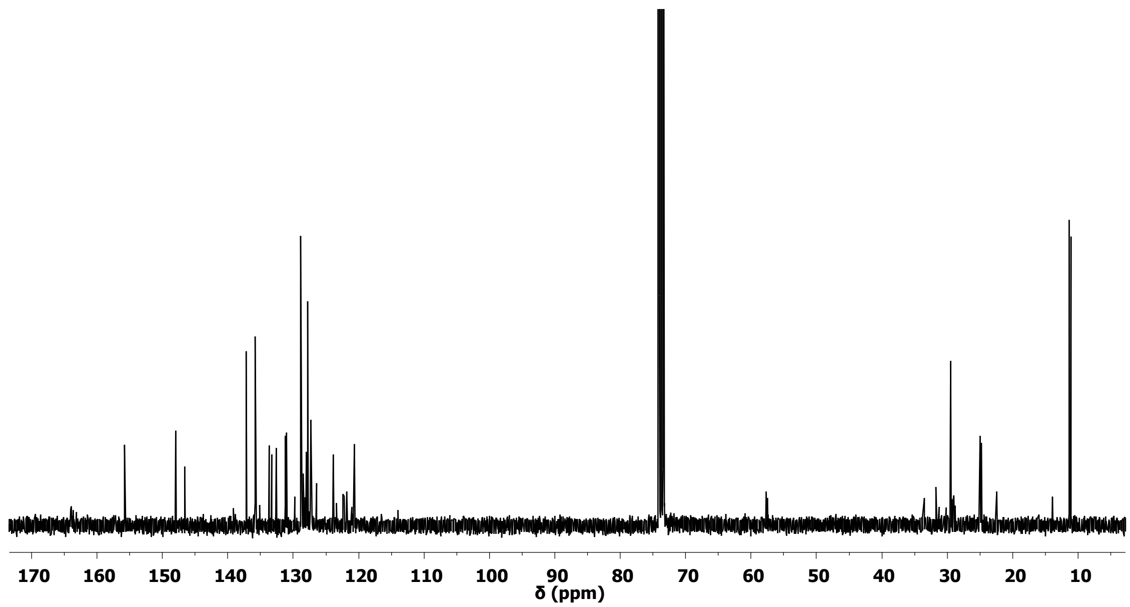

Figure S2. ^{13}C-RMN ($CDCl_2$-$CDCl_2$, 25°C) spectrum for **PDI 2**.

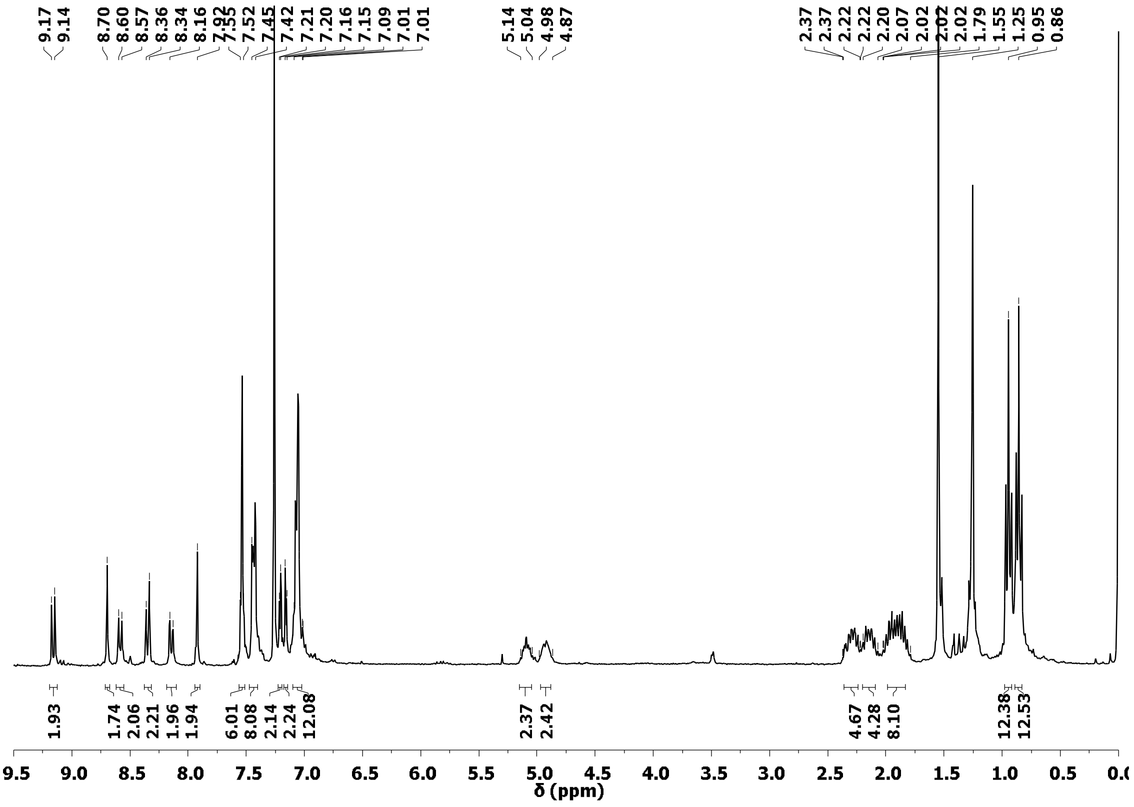

Figure S3. ^{1}H-RMN ($CHCl_3$, 25°C) spectrum for **PDI 3**.

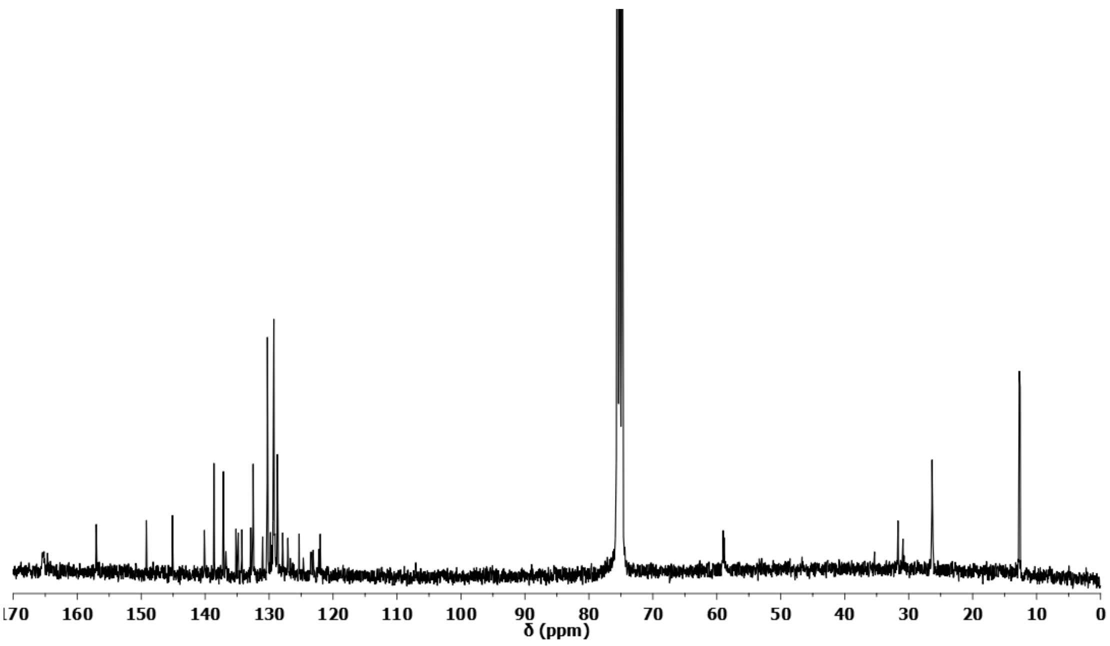

Figure S4. ^{13}C-RMN ($CDCl_2$-$CDCl_2$, 50°C) spectrum for **PDI 3.**

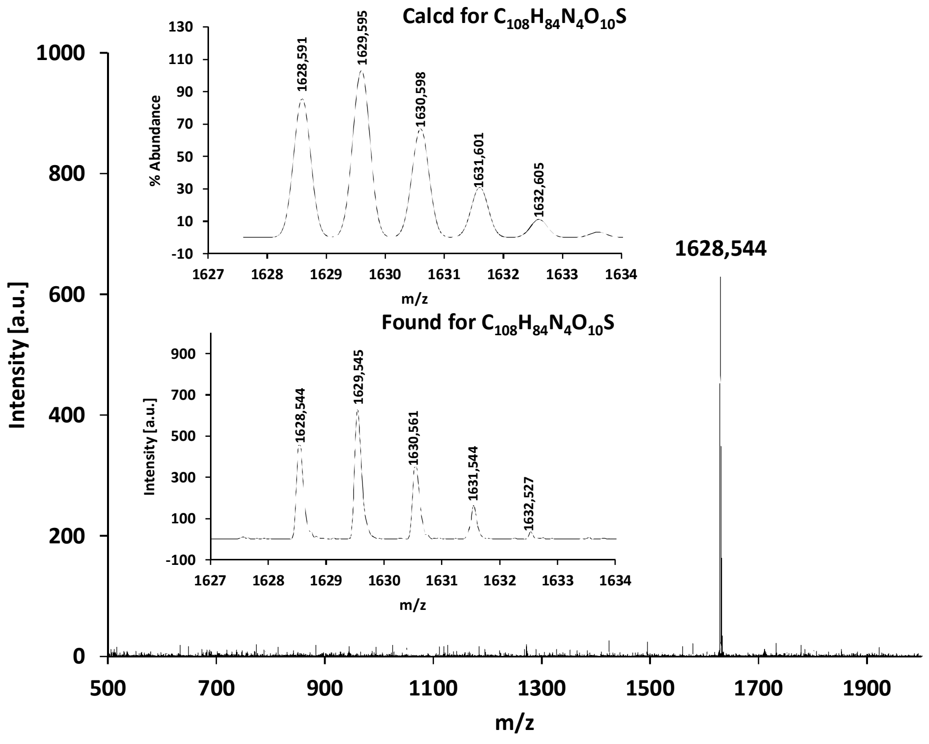

Figure S5. MALDI-TOF spectrum of **PDI 2**.

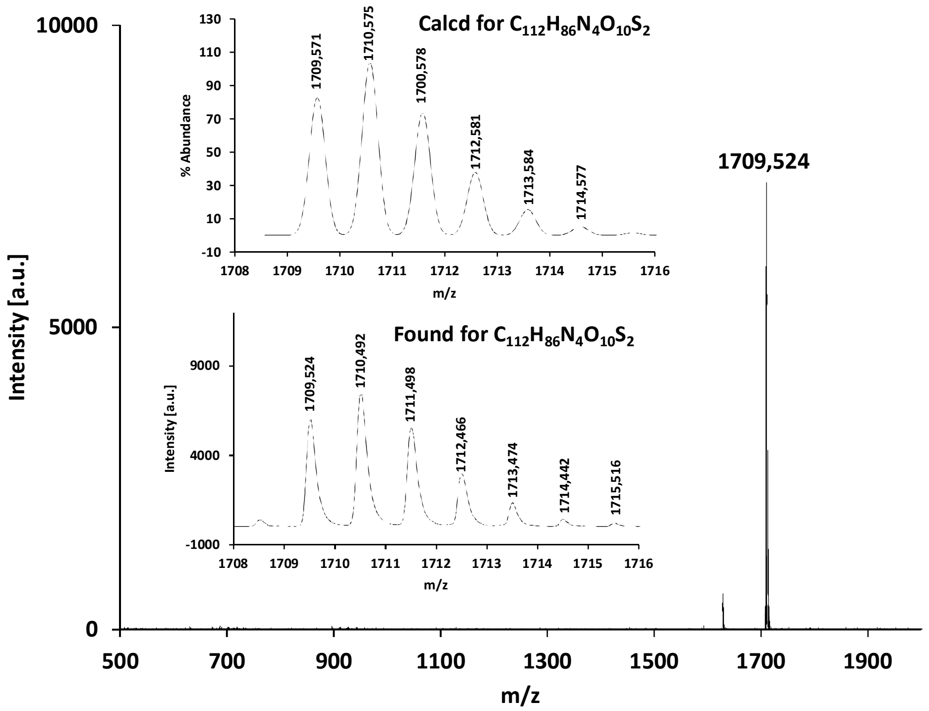

Figure S6. MALDI-TOF spectrum of **PDI 3**.

Solar cell fabrication

$PC_{71}BM$ was purchased from Solenne (Solenne BV, the Netherlands). High purity (HPLC gradient grade, 99.9 %) $CHCl_3$ was used for the active layer deposition. The solvent was dried with activated molecular sieves and kept in a sealed bottle with silver foil prior to use. Aluminium (99.999 %) and LiF (99.995 %) were purchased from Sigma-Aldrich. Prepatterned Indium Tin Oxide (ITO) 5 Ohm/square (PSiOTec, Ltd., UK) sodalime glass substrates were first rinsed with acetone to remove the residual photoresist layer. The substrates were placed in a Teflon holder and sequentially sonicated in acetone (1 × 10 min) and isopropanol (2 × 10 min), before finally being dried under a flow of nitrogen. The ITO substrates were ozone-treated in a UV-ozone cleaner for 30 mins in ambient atmosphere, and subsequently, in air, coated with a layer of filtered (0.45 µm, cellulose acetate) solution of Poly(3,4-ethylenedioxythiophene) : poly(styrenesulfonate) (PEDOT:PSS, HC Starck Baytron P) (4500 rpm 30 seconds followed by 3500 rpm 30 seconds). The PEDOT:PSS film was dried at 120 °C under an inert atmosphere for 15 min.

Active layers were spin-coated (1900 to 2600 rpm) in air over the PEDOT:PSS layer from a 25 or 15 mg/ml (total concentration) solution of the PTB7, and the corresponding PDI. The ratio between donor and acceptor is reported in Table S1. The cathode layer was deposited by thermal evaporation in an ultra high vacuum chamber ($1\cdot10^{-6}$ mbar). Metals were evaporated through a shadow mask leading to devices with an area of 9 mm^2. LiF (0.6 nm) and Al (100 nm) were deposited at a rate of 0.1 Å/s and 0.5-1 Å/s respectively. Following fabrication, the films were maintained under an atmosphere of nitrogen, and stored in the dark until used.

Table S1. Active layers deposition parameters

Device active layer	Ratio	Solvent	Conc. (total)	speed (rpm)	DIO (%)
PTB7:PDI 1	1:1	Chloroform	15	1900	0
PTB7:PDI 2	1:1	Chloroform	25	2200	0
PTB7:PDI 3	1:1	Chloroform	25	2600	0

Differential pulse voltammetry

DPV measurements were performed in a conventional three-electrode cell using a μ-AUTOLAB type III potentiostat/galvanostat at 298 K, over $CHCl_3$ and de-aerated sample solutions (~0.5 mM) containing 0.10 M tetrabutylammonium hexafluorophosphate ($TBAPF_6$) as supporting electrolyte. A carbon working electrode, $Ag/AgNO_3$ reference electrode and a platinum wire counter electrode were used. A ferrocene/ferrocenium couple was used as an internal standard for all measurements.

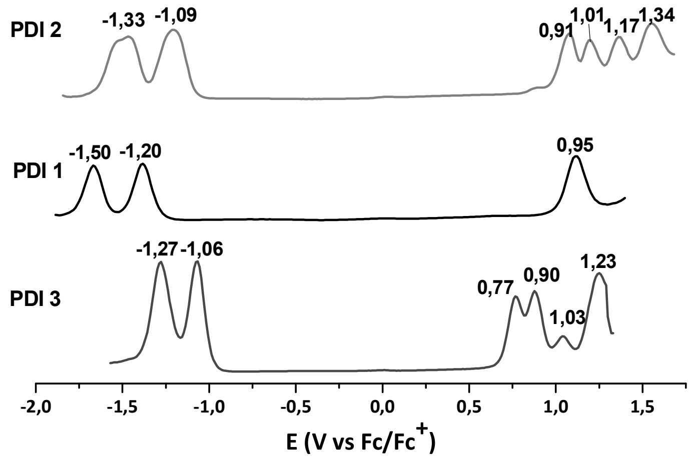

Figure S7. Differential pulse voltammetry of the PDI derivatives, recorded in chloroform with ferrocene as internal standard.

Table S2. Summary of the optical features derived from Absorption-emission spectroscopy and cyclic voltammetry.

Derivative	PDI 1	PDI 2	PDI 3
E_{HOMO} (eV)	-5.75	-5.71	-5.57
E_{LUMO} (eV)	-3.55	-3.71	-3.74
Band gap (eV)	2.20	2.00	1.83

Film and device characterisation

The UV-Vis absorption of films was measured using a Shimadzu UV-1700 spectrophotometer. The *J-V* characteristics of the devices were measured in a sealed capsule under an inert atmosphere, using a Sun 2000 Solar Simulator (150 W, ABET Technologies). The appropriate filters were utilized to simulate the AM 1.5G spectrum. The illumination intensity was measured at 100 mW m^2 using a calibrated silicon photodiode (NREL). The applied potential and cell current were measured with a Keithley 2400 digital source meter. The current-to-voltage (*J–V* curve) was plotted automatically with ad-hoc Labview© software. The IPCE (Incident Photon to Current conversion

Efficiency) was measured using a home made set up consisting of a 150 W Oriel Xenon lamp, a motorized monochromator, and a Keithley 2400 digital source meter. The photocurrent and irradiated light intensity were measured simultaneously and processed with additional Labview© software.

The thickness of the films was measured with a stylus profilometer Ambios Tech. XP-1, from a scratch sample made on the film.

AFM and X-Ray diffraction

AFM:

Atomic Force Microscopy (AFM) of the samples was performed in tapping mode on a Molecular Imaging model Pico SPM II (pico +). Images were collected in air using silicon probes with a typical spring constant of 1–5 nN/m, and at a resonant frequency of 75 kHz.

Table S3. Summary of the roughness values from the AFM topography micrographs from Figure 4.

	**PDI1**		**PDI2**		**PDI3**	
Image size	*PEAK TO PEAK*	*RMS*	*PEAK TO PEAK*	*RMS*	*PEAK TO PEAK*	*RMS*
1x1 μm	2,86	0,38	3,01	0,39	3,20	0,42
5x5 μm	6,52	0,44	8,38	0,43	4,72	0,44

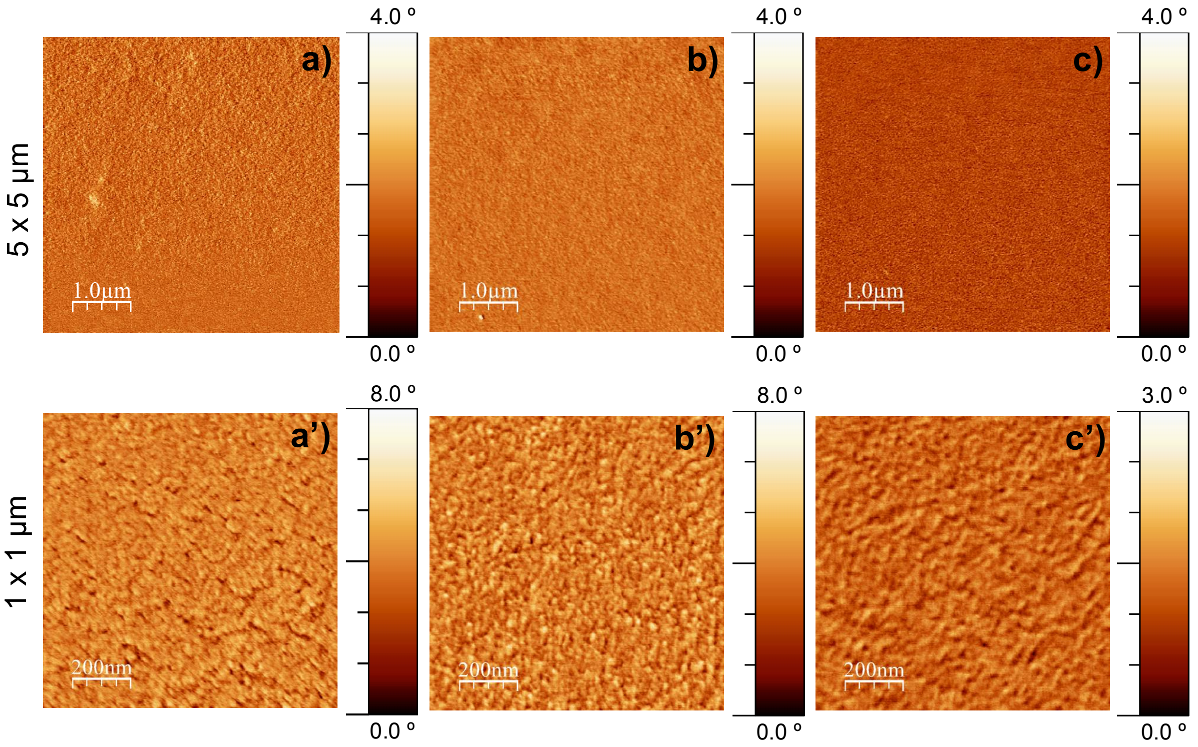

Figure S8. AFM phase images of the active layers made from PTB7 and PDI blends in conditions of optimized solar cell devices. a) and a') PTB7:PDI 1 (1:1); b) and b') PTB7:PDI 2 (1:1); c) and c') PTB7:PDI 3 (1:1).

<u>*XRD:*</u>

Conventional XRD measurements on thin films were carried out using a Bruker-AXS D8-Discover diffractometer with parallel incident beam (Göbel mirror), a vertical theta-theta goniometer, a XYZ motorized stage mounted on an Eulerian cradle, an incident and diffracted beam Soller slits, a 0.02° receiving slit, and a scintillation counter as a detector. Diffraction patterns were recorded over an angular 2theta range of 1° to 15°. The data were collected with an angular step of 0.05° at 10 sec per step. Cuk_{α} radiation was obtained from a copper X-ray tube operated at 40 kV and 40 mA.

2D XRD measurements were performed with the same diffractometer equipped with a HI-STAR area detector (multi-wire proportional counter of 30x30 cm with 1024x1024 pixel) and GADDS software (General Area Diffraction System). Samples were placed directly on the sample holder and the area of interest was selected with the aid of a video-laser focusing system. An X-ray collimator system allowed us to analyze areas with a width of

500 μm. 2D XRD patterns (one frame) were collected covering 1.2-22.3º 2θ from at a distance of 15 cm from the sample; exposition time was 600 sec per frame.

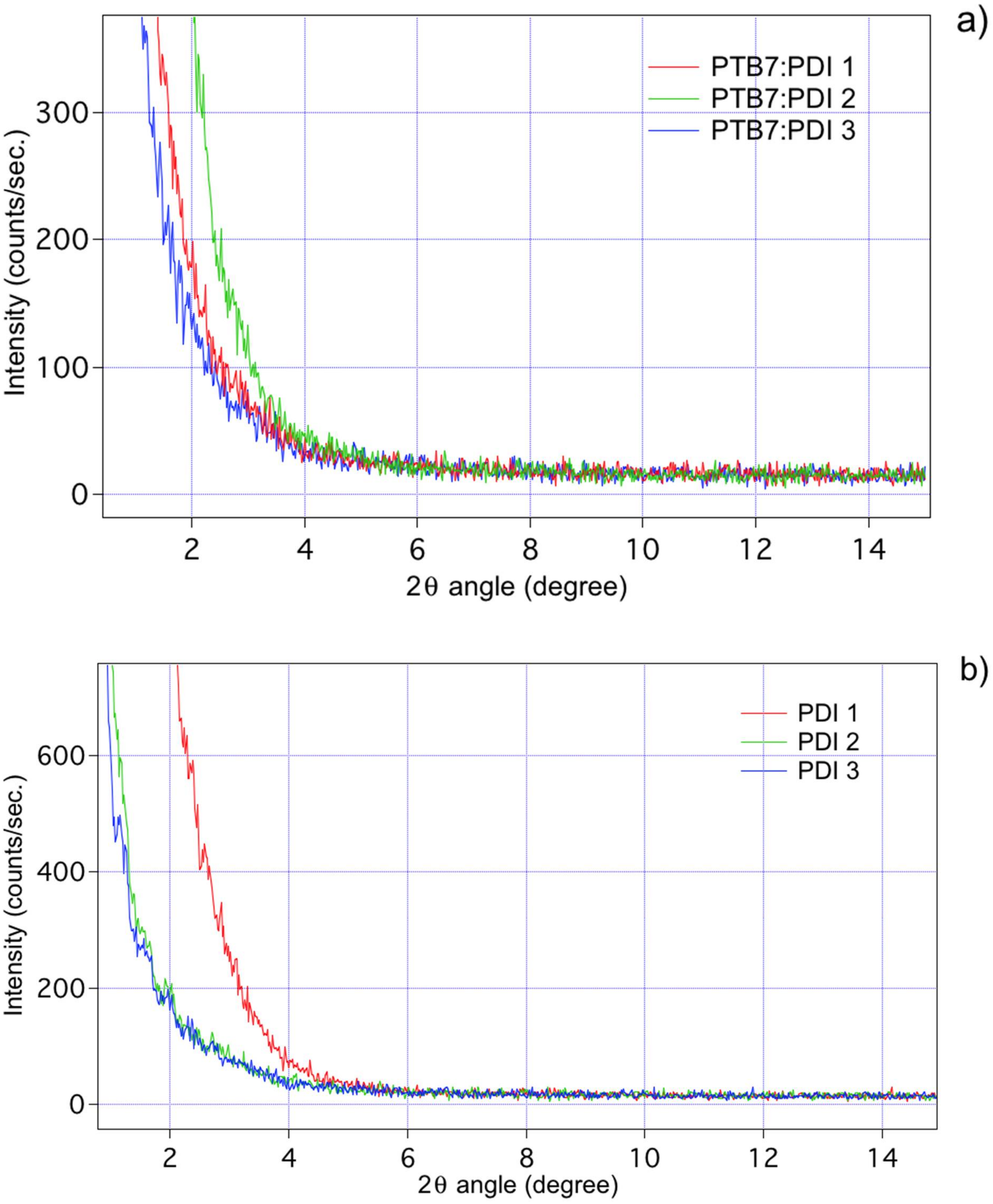

Figure S9. a) Bragg-Brentano point detector diffractograms of active layers deposited in identical conditions as those of optimized OSC devices. Bragg-Brentano point detector diffractograms of thin films of pristine PDIs acceptors deposited from chloroform (15mL/mg).

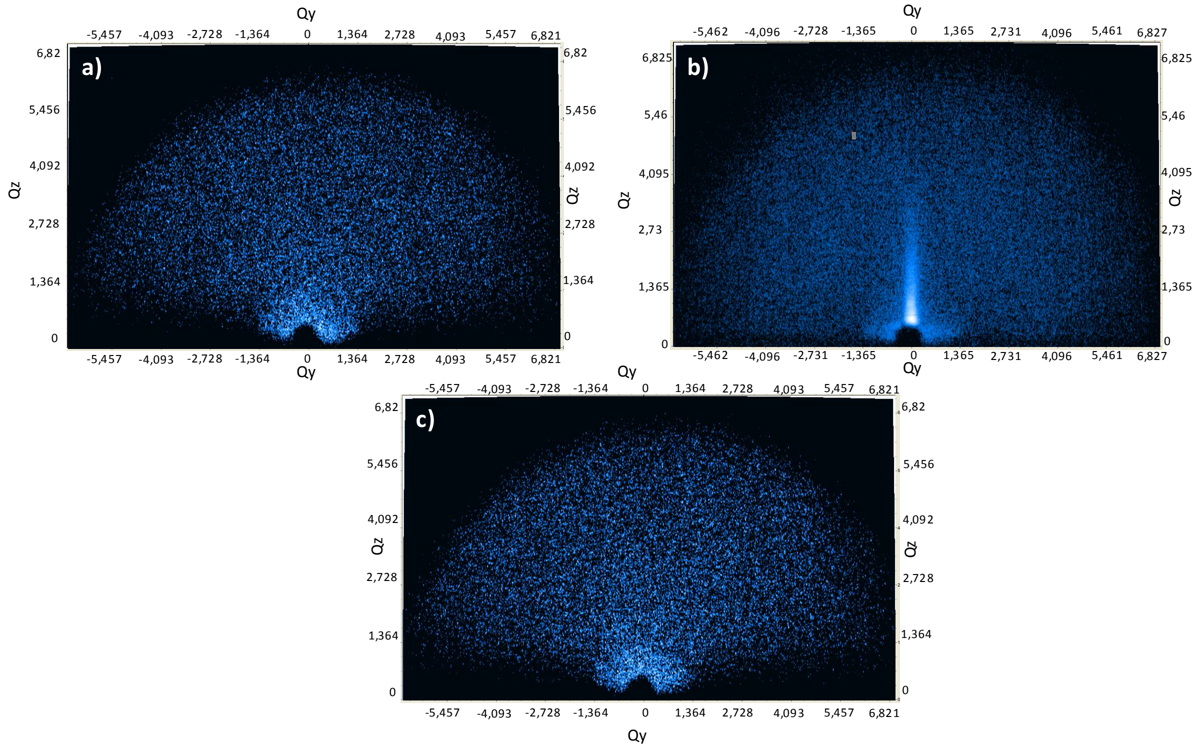

Figure S10. Out of plane GIXRD images of active layers. Recorded at a 30 cm distance from the samples with ω =0.5°, the incident beam angle with respect to the substrate, a) PTB7:PDI 1 b) PTB7:PDI 2 c) PTB7:PDI 3.

Hole and electron mobility:

Mobility measurements were performed using electron only devices which had ITO/ZnO/PTB7:Azafullerene/Al structures (Figure S11). Thicker metal cathodes (150 nm) were used to aid cooling (in order to prevent damage associated with device heating when measuring at high voltages). Mobility values were obtained by fitting the obtained J-V plots in the SCLC region to a Mott-Gurney as in equation (1) with the static permittivity of the active layer (ε) fixed to 3, μ_0 being the zero-field mobility.

$$J=9/8\ (\varepsilon\mu_0)/L^3 \times V^2 \qquad (1)$$

Mobility measurements were repeated at least three times (using different devices) to confirm the reproducibility of the results. High electron mobility Zinc Oxide was deposited *via* a sol-gel.

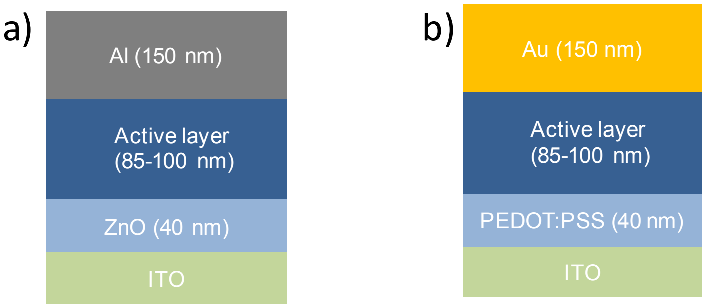

Figure S11. Device architecture used for the electron-only devices.

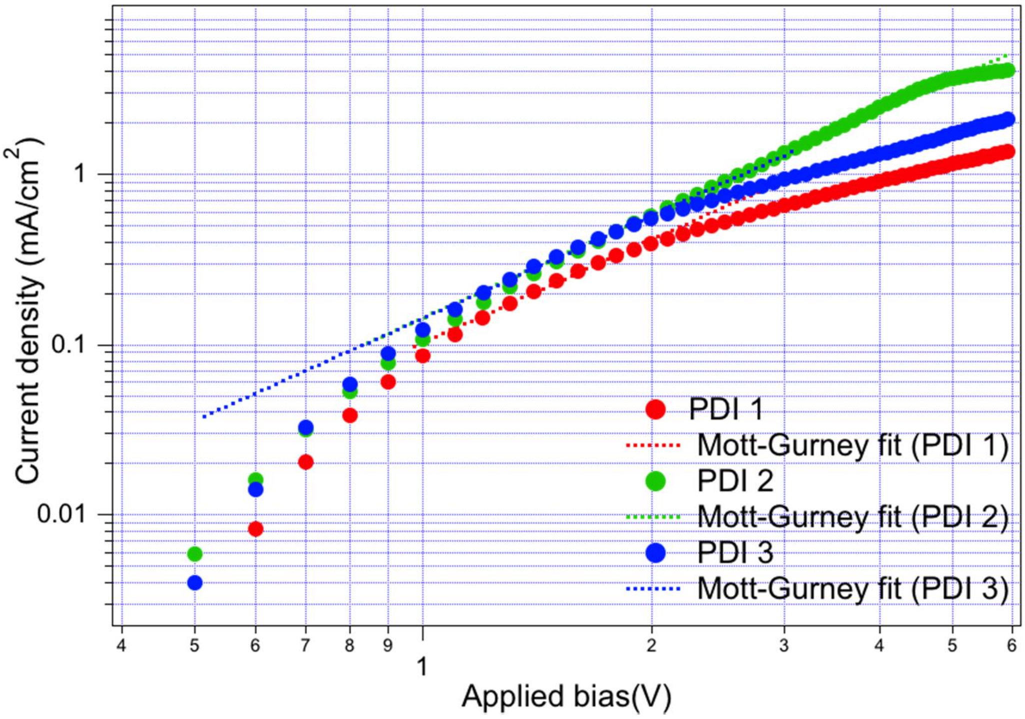

Figure S12. Hole-only devices *J-V* characteristics measured in the dark.

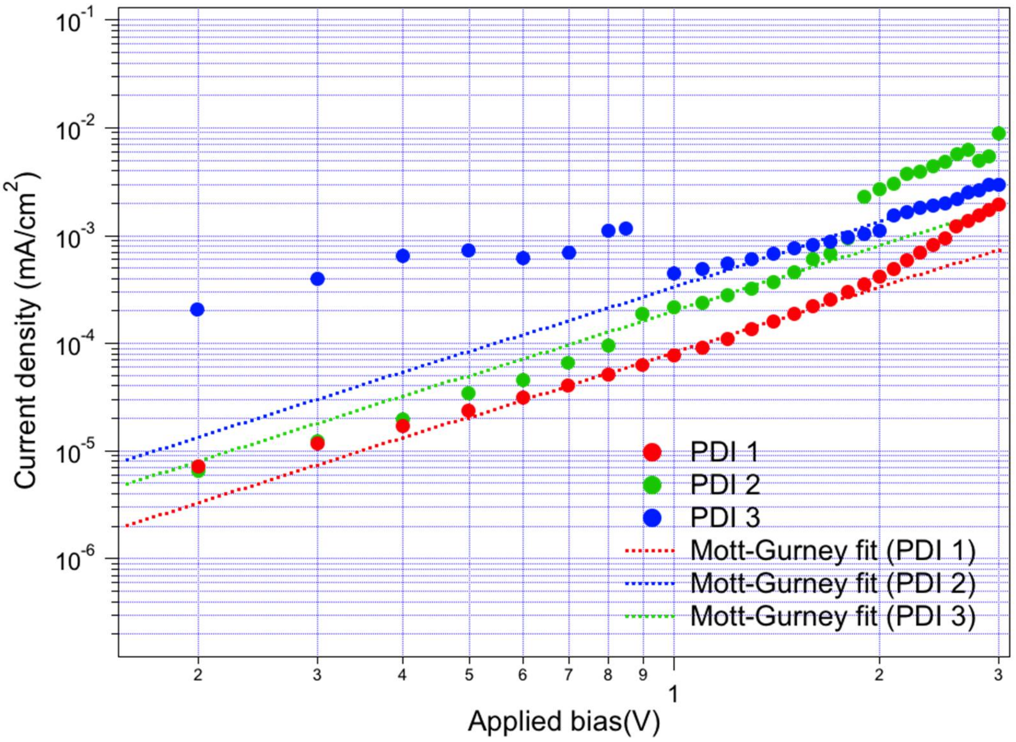

Figure S13. Electron-only devices' *J-V* characteristics measured in the dark.

CE/TPV analysis

Charge extraction (CE) was employed to probe the charge density within the active layer of the device under working conditions using an ad hoc system. Devices were held at open circuit by applying bias from a focused array of LEDs. Once the device reached a steady state it was then short-circuited with LEDs simultaneously switched off (switch-off time / relay = 300 ns), leaving the charge stored in the active layer to decay through a small 50 Ω resistor. A Yokogawa 2052 digital oscilloscope was used to record the voltage decay across the resistor. Using Ohm's law, the voltage transient could be turned into a current transient which was subsequently used to calculate the total charge in the active layer at each light applied bias. In general, the device produced open circuit voltage values corresponding to >1 Sun conditions to 0 V.

Transient photovoltage (TPV) measurements were carried out on working devices through the application of a light bias (the same ring of LEDs used in CE), and by holding the device in a steady state in open circuit conditions. Once the device reached steady state conditions, a low-

intensity laser pulse (PTI GL-3300 Nitrogen Laser) irradiated the sample to allow a small excess number of charge carriers to be generated. As the device was being held at open-circuit, the excess charge generated inevitably recombined. The transient decay of the charge carriers was recorded using a Yokogawa 2052 digital oscilloscope. Sweeping from high-applied bias (high illumination) to low-applied bias (low illumination) allowed a correlation between charge carrier lifetime and voltage to be made. The irradiation wavelength was chosen to be close to, but not matching, the maximum of the donor absorption spectrum. A graded neutral density filter was used to control the intensity of the small perturbation, usually keeping the value at between 5 and 10 mV.

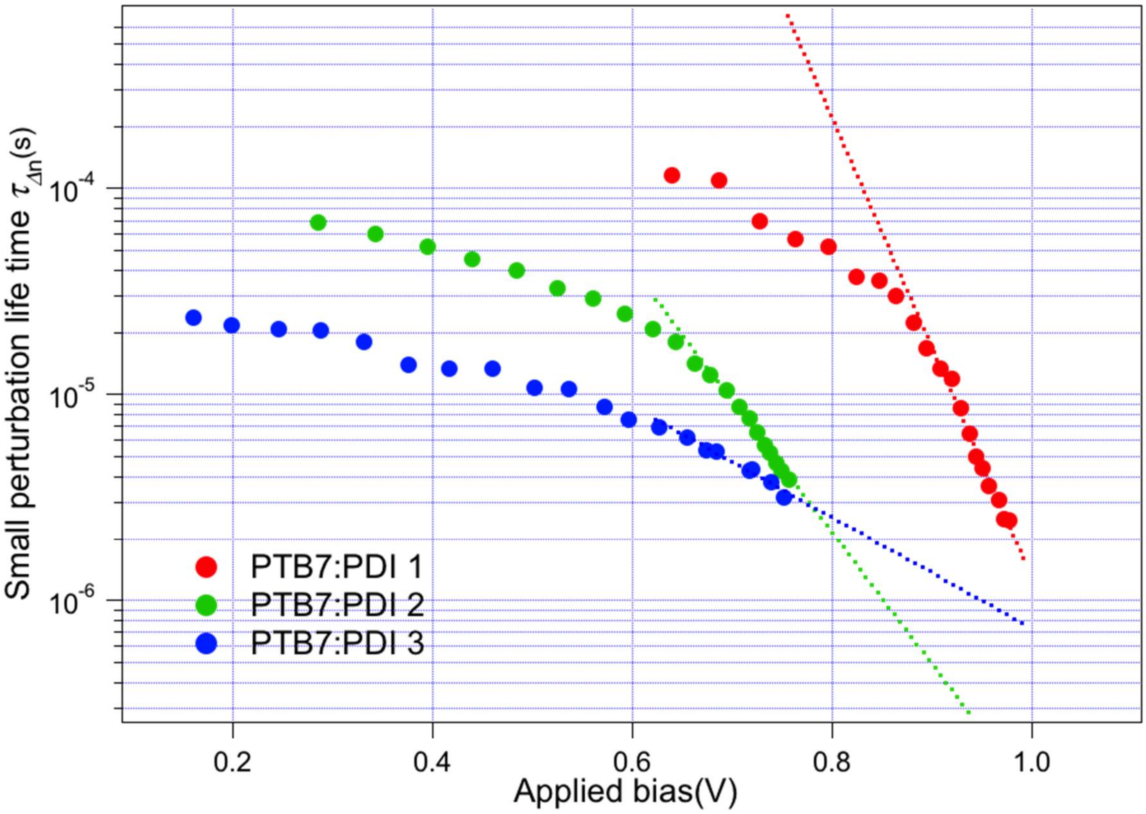

Figure S14. Carriers life time ($\tau\Delta n$) versus open circuit voltage (V_{OC}) plot. Curves are fitted to exponential decays of the form $\tau\Delta n = e^{-\beta V_{oc}}$.

PTB7/PC70BM solar cell

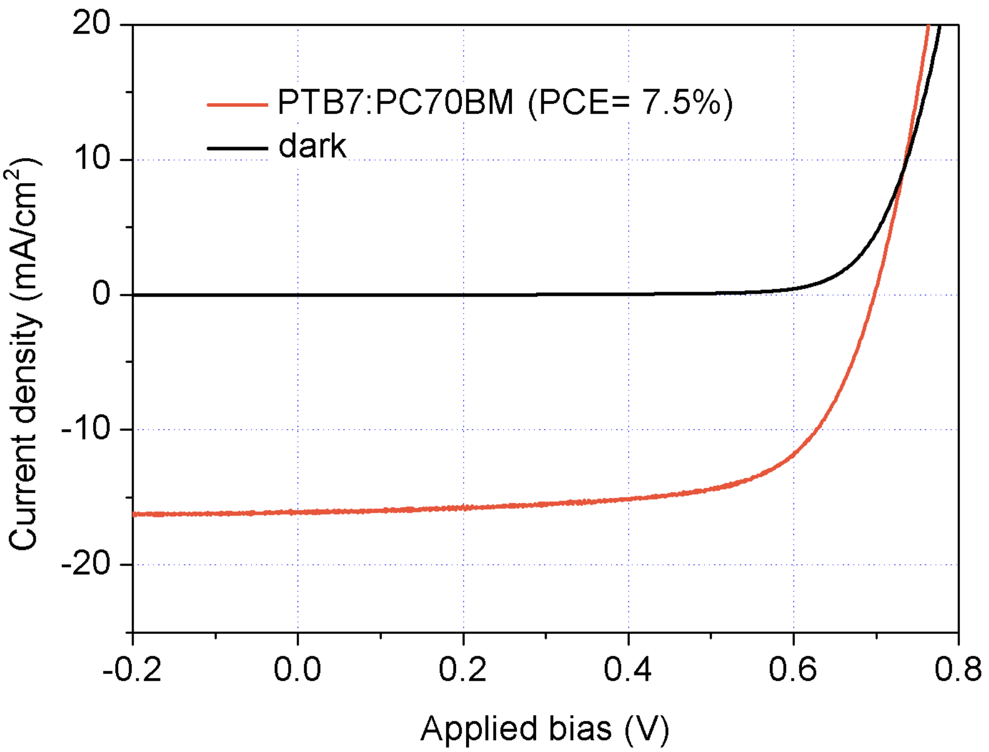

Figure S15: *J-V* characteristics of a typical PTB7:PC70BM solar cells fabricated in identical conditions as those used for PDI-based devices (ITO/PEDOT:PSS/PTB7:PC70BM/Ca/Ag).
